# OSlihc: An Online Prognostic Biomarker Analysis Tool for Hepatocellular Carcinoma

**DOI:** 10.3389/fphar.2020.00875

**Published:** 2020-06-10

**Authors:** Yang An, Qiang Wang, Guosen Zhang, Fengjie Sun, Lu Zhang, Haojie Li, Yingkun Li, Yanyu Peng, Wan Zhu, Shaoping Ji, Xiangqian Guo

**Affiliations:** ^1^Department of Predictive Medicine, Institute of Biomedical Informatics, Cell Signal Transduction Laboratory, Bioinformatics Center, Henan Provincial Engineering Center for Tumor Molecular Medicine, Kaifeng Key Laboratory of Cell Signal Transduction, School of Basic Medical Sciences, School of Software, Henan University, Kaifeng, China; ^2^Department of Anesthesia, Stanford University, Stanford, CA, United States

**Keywords:** liver hepatocellular carcinoma, prognosis biomarker, survival analysis, gene expression profiling, survival outcome

## Abstract

Liver hepatocellular carcinoma (LIHC) is one of the most common malignant tumors in the world with an increasing number of fatalities. Identification of novel prognosis biomarker for LIHC may improve treatment and therefore patient outcomes. The availability of public gene expression profiling data offers the opportunity to discover prognosis biomarkers for LIHC. We developed an online consensus survival analysis tool named OSlihc using gene expression profiling and long-term follow-up data to identify new prognosis biomarkers. OSlihc consists of 637 cases from four independent cohorts. As a risk assessment tool, OSlihc generates the Kaplan–Meier survival plot with hazard ratio (HR) and p value to evaluate the prognostic value of a gene of interest. To test the reliability of OSlihc, we analyzed 65 previous reported prognostic biomarkers in OSlihc and showed that all of which have significant prognostic values. Furthermore, we identified four novel potential prognostic biomarkers (*ATG9A*, *WIPI1*, *CXCL1*, and *CSNK2A2*) for LIHC, the elevated expression of which predict the unfavorable survival outcomes. These genes (*ATG9A*, *WIPI1*, *CXCL1*, and *CSNK2A2*) may be potentially new biomarkers to identify at-risk LIHC patients when further validated. By OSlihc, users can evaluate the prognostic abilities of genes of their interest, which provides a platform for researchers to identify prognostic biomarkers to further develop targeted therapy strategies for LIHC patients. OSlihc is public and free to the users at http://bioinfo.henu.edu.cn/LIHC/LIHCList.jsp.

## Introduction

As one of the most common tumors, hepatocellular carcinoma (HCC) also known as liver hepatocellular carcinoma (LIHC), is a leading cause of death of cancer globally (Yu et al., 2010; Pinato et al., 2014; Cristea et al., 2015). A number of staging and grading systems have been commonly used for LIHC, including TNM, Barcelona Clinic Liver Cancer (BCLC), albumin-bilirubin (ALBI) grading, and Okuda system (Selcuk, 2017). For LIHC patients, surgical excision is an optimal treatment (Mullath and Krishna, 2019). However, the majority of LIHC patients have the risks of recurrence and metastasis, leading to a much lower 5 years survival (Buonaguro et al., 2015). To improve patients’ quality of life, treatment should be tailored according to more accurate predicted disease outcome (Pinato et al., 2014). So far, some biomarkers have been identified to provide insights of disease outcomes. For example, α-fetoprotein (AFP) and des-gamma-carboxy-prothrombin (DCP), PCNA and Ki-67 are the most frequently used biomarkers for LIHC (King et al., 1998; Lee et al., 2013; Lin et al., 2015; Lee et al., 2016; Ma et al., 2016; Yao et al., 2016). However, due to the high heterogeneity of LIHC, more prognosis biomarkers for LIHC need to be discovered.

In the present study, we developed an easy-to-use web server named OSlihc, to provide an online platform to evaluate potential prognostic biomarkers for LIHC in multiple independent cohorts. In addition, we have validated the prognostic ability of 65 previously reported biomarkers using this web tool, and have identified four novel potential prognostic biomarkers for LIHC, including *ATG9A*, *WIPI1*, *CXCL1*, and *CSNK2A2*. By using OSlihc, researchers and clinicians can expediently evaluate the prognostic value of the genes of their interests.

## Methods

### Data Collection and Processing

Gene expression profiling data and clinical follow-up information of hepatocellular carcinoma were collected from TCGA (The Cancer Genome Atlas) and GEO (Gene Expression Omnibus) database. For TCGA dataset, gene expression profiling (RNA-seq, level-3, HiSeqV2) and follow-up data (361 cases) were downloaded in 2018 (**Table 1**). For GEO datasets, the keywords, including “hepatocellular carcinoma”, “gene expression” and “survival” were used to search in GEO database. Then the datasets containing mRNA expression and survival data with at least 50 cases were included. As a result, three GEO datasets (GSE76427, GSE20140, and GSE27150) with 115, 80, and 81 clinical LIHC cases, respectively (**Table 1**), were collected.

**Table 1:** Summary of datasets in OSlihc.

Dataset	Sample size	Data type	Platform	No. of death	Median of OS (months)	Survival terms
TCGA	361	RNA-seq	Illumina HiSeqV2	129	20.03	OS, DSS, DFI, PFI
GSE76427	115	cDNA array	GPL10558	23	13.92	OS, RFS
GSE20140	80	cDNA array	GPL5474	32	96.15	OS
GSE27150	81	cDNA array	GPL13128	28	39	OS
Total	637			212	22.43	

Five clinical survival terms, including OS (overall survival), DSS (disease-specific survival), RFS (relapse free survival), DFI (disease-free interval), and PFI (progression-free interval) (Liu et al., 2018), were used in OSlihc web server (**Table 1**). The clinicopathologic characteristics of LIHC patients in OSlihc database are summarized in **Tables S1** and **S2**.

### Development of OSlihc

**Figure 1:**
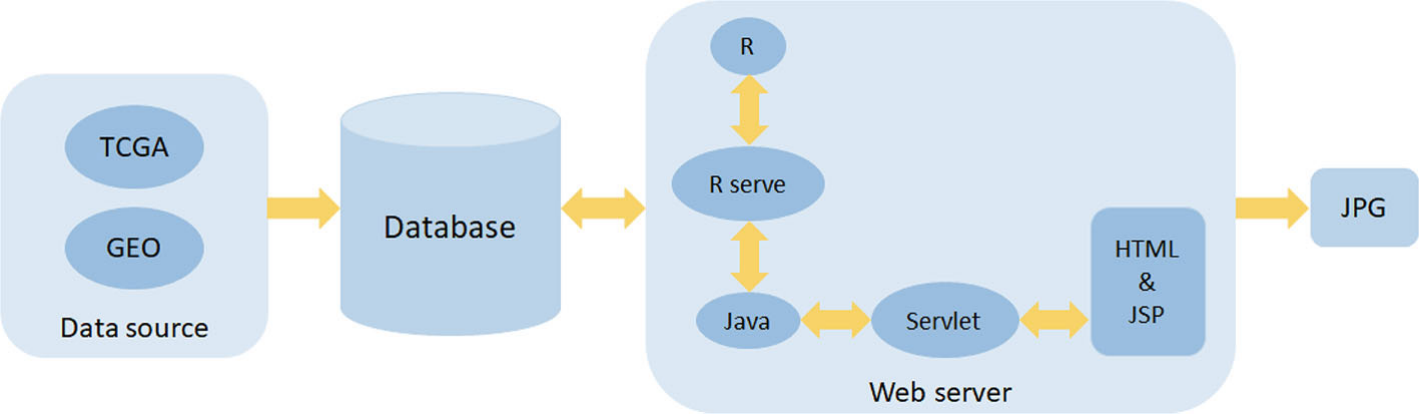
Flowchart of web server architecture.

OSlihc includes two major assemblies: data storage and analysis. The OSlihc web server is hosted in a tomcat 7.0 server in Windows 2008 system, the web interface is developed by HTML and JSP languages, the controlling part is developed by Servlet, and the database is handled by SQL Server which stores and manages the gene expression profiling and clinical data. The Servlet acts as a connecting layer between the frontend and the backend to provide interaction to users. Moreover, OSlihc web server is operated by R and Java, which manipulates the request from users and then returns the analysis results to users, the R package “Rserve” acts as a connecting layer between R and Java. The JDBC package serves as the connection middleware between R, Java, and SQL. In addition, the R package “survival” generates Kaplan–Meier survival curves and calculates hazard ratio with 95% confidence intervals and *p* value. System architecture flow diagram is presented in **Figure 1**, as previous described (Wang et al., 2019a; Wang et al., 2019b; Xie et al., 2019; Zhang et al., 2019). OSlihc is available at http://bioinfo.henu.edu.cn/LIHC/LIHCList.jsp.

### Validation of Prognostic Biomarkers in OSlihc

To assess the performance and reliability of prognostic analysis of OSlihc web server, previously reported prognostic biomarkers for LIHC were searched in PubMed using the keywords “hepatocellular carcinoma”, “survival”, “prognosis”, and “biomarker”. As a result, we collected 67 papers with 65 reported prognostic biomarkers. The prognostic capabilities of these reported prognostic genes were evaluated by plotting the survival curve in OSlihc. We input gene symbols of the 65 reported prognostic biomarkers into the “Gene symbol” box individually, and then clicked the “Kaplan–Meier plot” button to obtain the survival curve plot. We listed “clinical survival terms”, “cut-off”, “case”, “p value”, “HR”, “95%CI”, “detection level”, and “validation”, and compared the “prognostic outcome” between “In OSlihc” and “In reference” when the reported prognostic gene exhibited the higher expression level.

### Identification of Novel Potential Prognostic Biomarkers in OSlihc

To identify prognostic biomarker candidates for LIHC, we genome-widely evaluated the prognostic values of human genes for each dataset using Cox regression analysis. Genes significantly related to prognosis were selected from each dataset (cox p value < 0.05). Then these genes were overlapped among the four datasets, as a result, four genes were identified as potential prognostic biomarkers for they were significantly related to prognosis in three datasets, including *ATG9A*, *WIPI1*, *CXCL1*, and *CSNK2A2*.

## Results

### Clinicopathologic Characteristics of LIHC Patients in OSlihc

**Figure 2:**
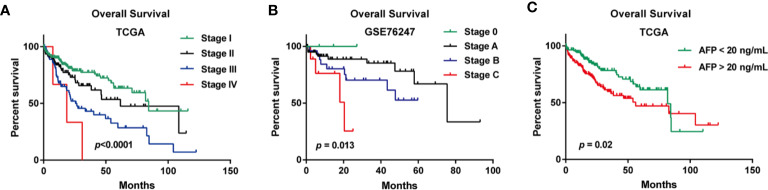
Survival analysis of clinical characteristics of the patients included in OSlihc. **(A)** Pathologic stage, **(B)** BCLC stage, **(C)** Serum AFP level.

In the TCGA cohort, the median age of a total of 361 LIHC patients was 61. When examining the disease stages of LIHC patients, stage I patients accounts for 46% of all the LIHC patients (n=167), stage II accounts for 23% (n=82), stage III accounts for 23% (n=84), and stage IV accounts for 1% (n=4) (**Table S1**). In the GSE76427 cohort, the median age of a total of 115 patients was 64, and the distribution of BCLC stage was that stage A accounts for 64% (n=74), stage B accounts for 24% (n=28), stage C accounts for 8% (n=9), and stage 0 accounts for 3% (n=4) (**Table S2**). In the GSE20140 cohort, microvascular invasion was present in 21% (n=17) of LIHC patients, and absent in 56% (n=45) of LIHC patients (**Table S2**). The clinicopathologic information of GSE27150 dataset were unavailable. The median OS time is 22.43 months of all LIHC patients in OSlihc (**Table 1**). The summaries of clinicopathologic characteristics of each cohort were presented in **Tables S1** and **S2**. The Kaplan–Meier plots for LIHC patients in OSlihc stratified by different pathologic stage and BCLC stage were presented in **Figure 2**. The pathologic stage and BCLC stage were significantly associated with OS (*p*<0.0001 and *p*=0.013, respectively) (**Figures 2A, B**). And the serum AFP level was also significantly associated with OS (*p*=0.02) (**Figure 2C**).

### Application of OSlihc

**Figure 3:**
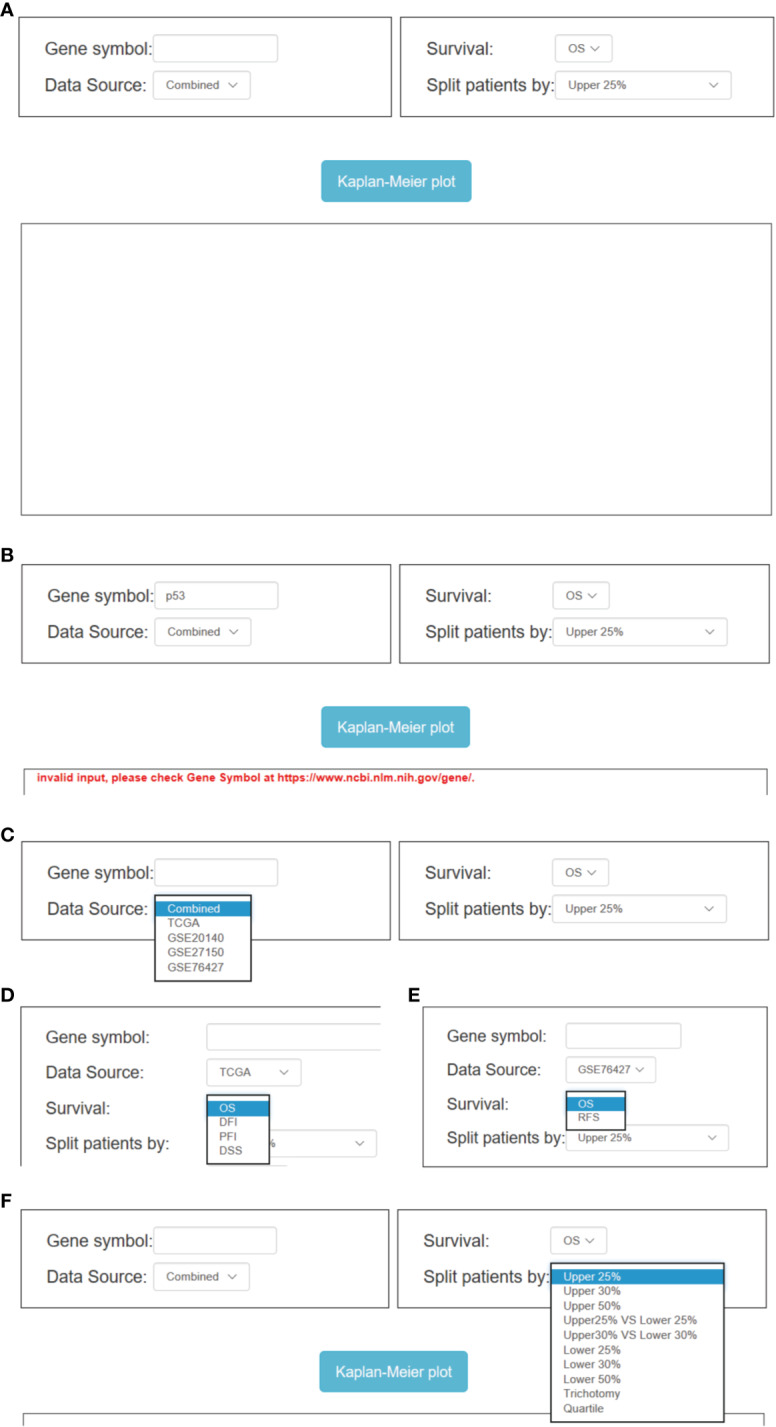
Overview of OSlihc. **(A)** Screenshot of OSlihc main interface. **(B–F)** Input and output interface of OSlihc.

OSlihc has the ability of drawing a forest plot for all the cohorts to highlight the prognostic significance of interested genes (cutoff: upper 50% vs. lower 50%). In OSlihc, “Gene symbol”, “Data Source”, “Survival”, and “Split patients by” are the four main parameters to evaluate the prognostic value of a gene of interest in LIHC (**Figure 3A**). In general, the official gene symbol is required in the “Gene symbol” box by the users. A red warning message would be shown if the input was not an official gene symbol (**Figure 3B**). Drop-down menu of “Data source” provides five options for users to take each of the four individual cohorts (TCGA, GSE76427, GSE20140, and GSE27150 datasets) or a combined cohort pooling all four above mentioned cohorts into one for survival analysis (**Figure 3C**). The combined cohort denotes that each cohort was individually stratified into subgroups by choosing a cutoff value for gene expression level, which were then pooled together for prognosis analysis. As a result, users have the choice to assess the prognostic value of a candidate gene in a single or the combined cohort according to the needs. In OSlihc, five survival terms could be generated, including OS, DSS, RFS, DFI, and PFI. OS could be measured in all the cohorts and in combined cohort, while RFS could be calculated only in GSE76427, DSS, DFI, and PFI could be analyzed only in the TCGA cohort (**Figures 3D, E**). “Split patients by” option could categorize LIHC patients into 2–4 subgroups by the expression level of the inputted gene (such as Upper or Lower 25%, 30%, 50%), users can select the different cutoff values (**Figure 3F**).

In addition, additional multiple optional parameters could also be set as confounding clinical factors to sub-categorize LIHC patients for users to choose, including “Gender”, “BCLC Stage”, “TNM”, “Grade”, “Alcohol”, “Race”, etc. (**Figure S1**). Take “BCLC Stage” (on the interface of GSE76427 cohort) for example, users can select BCLC Stage (All, A, B, C, or 0) of LIHC from the drop-down menu box in OSlihc to evaluate the BCLC Stage-specific prognostic value of putative genes (**Figure S1C**). When all the options have been set, user could click the “Kaplan–Meier plot” button, OSlihc will receive the user’s request and return the prognosis analysis results for the inputted gene to users on the output web page, which graphically displays the Kaplan–Meier survival curve with p value and HR (with 95% confidence interval).

### Validation of Previously Reported LIHC Prognostic Biomarkers in OSlihc

**Figure 4:**
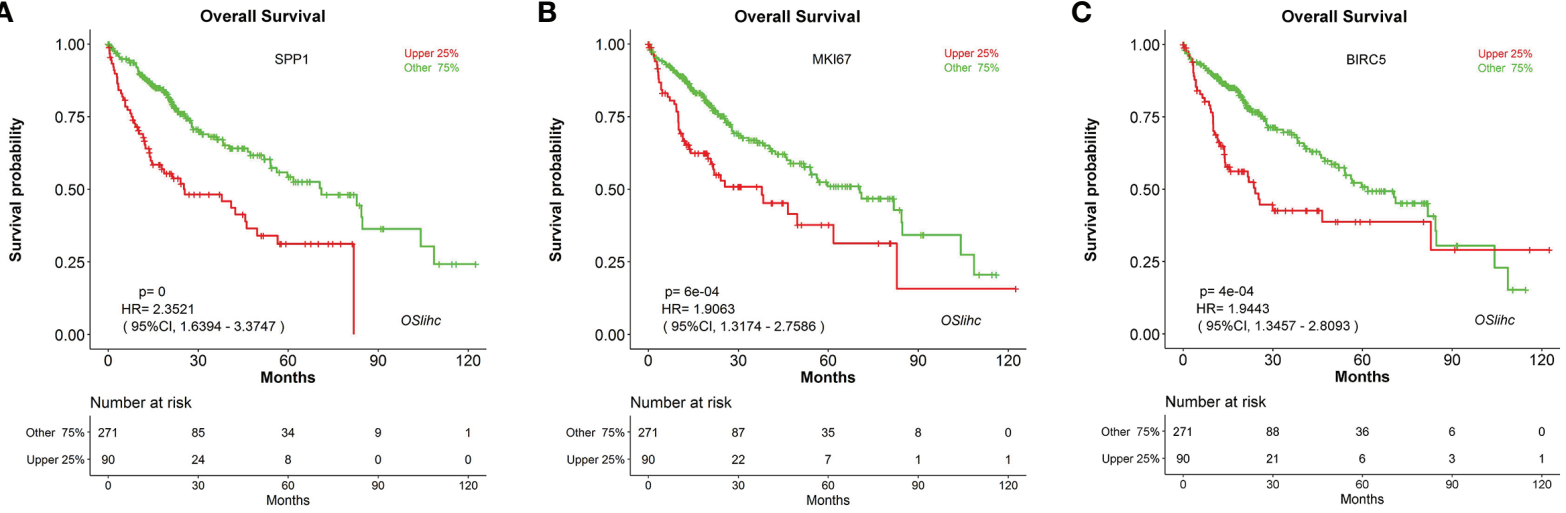
Validation of the top three high-frequency reported biomarkers in OSlihc. Kaplan–Meier plots for **(A)** *SPP1*, **(B)** *MKI67*, and **(C)** *BIRC5* in terms of OS. *p*-value, confidence interval (95%CI) and number at risk are as shown. The y-axis represents survival rate and the x-axis represents survival time (months). *p* = 0 denotes *p* < 0.0001.

In order to assess the reliability and capability of OSlihc web server in performing prognosis analysis, we collected 65 previously reported prognostic biomarkers at protein, mRNA or serum levels from 67 literatures. CDK1, HDAC2, RORA, FOXO3, and PCNA were among these 65 genes to be evaluated in OSlihc. The analysis results showed that all the 65 previously reported prognostic biomarkers have been demonstrated significant prognostic abilities (not only OS, but also DSS, DFI, and PFI) in OSlihc web server (**Table 2** and **Table S3**). As previously described (Ouyang et al., 2016), *SPP1*, *MKI67*, and *BIRC5* genes are significantly correlated with survival (OS, DSS, DFI, and PFI) in OSlihc (**Table 2**, **Figure 4**, and **Figure S2**). Patients with higher *SPP1*, *MKI67*, or *BIRC5* expression have shorter survival (*p*<0.0001, *p*=6e-04 or 4e-04, respectively), while patients with lower *SPP1*, *MKI67*, or *BIRC5* expression exhibited longer survival (**Table 2** and **Figure 4**). When compared with normal tissue, *SPP1*, *MKI67*, or *BIRC5* expression was significantly increased in LIHC tissues (**Figure S3**).

**Table 2:** Validation of previously published prognostic biomarkers for LIHC in OSlihc.

Gene symbol	Biomarker name	Clinical survival terms	In OSlihc					In reference					Prognostic outcome (higher expression)	Ref.
			Cut-off	p value	HR	95%CI	Case	Cut-off	p value	case	Detection level	Validation		
SPP1	Osteopontin (OPN)	OS	Upper 25%	0	2.3521	1.6394-3.3747	361	/	<0.05	110	serum	Yes, serum biochemical assay	worse	(Yang et al., 2008; Jin et al., 2014; Qin, 2014)
		DSS		0.0126	1.8422	1.1403-2.9763			/			
		DFI		0.0183	1.5	1.071-2.1008			DFS: p < 0.05			
		PFI		0.0078	1.5611	1.1242-2.1679			/			
BIRC5	Survivin	OS	Upper 25%	4e-04	1.9443	1.3457-2.8093	361	upper n = 31/lower n = 41	/	72	protein	Yes, IHC assay	worse	(Fields et al., 2004)
		DSS		2e-04	2.4062	1.5199-3.8095			/			
		DFI		9e-04	1.753	1.2585-2.4419			DFS: 0.0098			
		PFI		0.0012	1.7165	1.2375-2.3809			/			
MKI67	Ki-67	OS	Upper 25%	6e-04	1.9063	1.3174-2.7586	361	upper n = 47/lower n = 0	0.0009	67	protein (Ki-67 labeling index >10% indicated poor prognosis)	Yes, IHC assay	worse	(King et al., 1998)
		DSS	0.0011		2.1783	1.3664-3.4729			/			
		DFI	0.002		1.6821	1.2089-2.3404			DFS: 0.02			
		PFI	0.0027		1.6475	1.1891-2.2827			/			

### Identification of Novel Potential LIHC Prognostic Biomarkers in OSlihc

**Figure 5:**
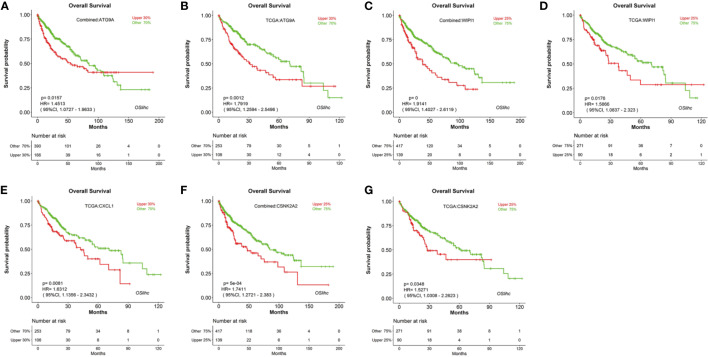
Evaluation of the prognostic values of potential biomarkers in OSlihc. Kaplan–Meier plots for high (red) and low (green) *ATG9A*, *WIPI1*, *CXCL1*, or *CSNK2A2*-expression cohort in combined dataset **(A, C**, **F)** and TCGA dataset **(B, D, E**, **G)**. *p*-value, confidence interval (95%CI) and number at risk are as shown. The y-axis represents survival rate and the x-axis represents survival time (months).

To identify new prognostic biomarker candidates for LIHC, we genome-widely evaluated the prognostic values of human genes using Cox regression analysis, and identified four genes which show significant association with survival in OSlihc, and these four genes including *ATG9A* (Tang et al., 2013), *WIPI1* (D’Arcangelo et al., 2018), *CXCL1* (Zou et al., 2014; le Rolle et al., 2015; Nakashima et al., 2015; Wang et al., 2016; Cabrero-de Las Heras and Martinez-Balibrea, 2018; Zhuo et al., 2018), and *CSNK2A2* have not been reported to exhibit the prognostic values in LIHC up to now and were subject to the prognosis analysis in OSlihc (**Table S4**). Notably, all these four genes exhibited good performance in predicting LIHC patient outcome (**Table 3** and **Figure 5**). Moreover, we found that patients with higher *ATG9A*, *WIPI1*, *CXCL1*, or *CSNK2A2* expression have the worst survival rate (**Figure 5**), indicating that the elevated expression of all these potential predictors could predict the unfavorable prognosis.

**Table 3:** Evaluation of novel potential prognostic biomarkers for LIHC in OSlihc.

Gene symbol	In OSlihc					case
	cut-off	survival terms	p value	HR	95%CI	
ATG9A	upper 30%	OS	0.0157	1.4513	1.0727–1.9633	556
		OS	0.0012	1.7919	1.2594–2.5496	361
		DSS	0.0246	1.6859	1.0693–2.6581	
		DFI	no significance			
		PFI	no significance			
WIPI1	upper 25%	OS	<0.0001	1.9141	1.4027–2.6119	556
		OS	0.0176	1.5866	1.0837–2.323	361
		DSS	no significance			
		DFI	no significance			
		PFI	no significance			
CXCL1	upper 30%	OS	no significance			636
		OS	0.0081	1.6312	1.1356–2.3432	361
		DSS	0.0454	1.6077	1.0098–2.5595	
		DFI	no significance			
		PFI	no significance			
CSNK2A2	upper 25%	OS	5e-04	1.7411	1.2721–2.383	556
		OS	0.0348	1.5271	1.0308–2.2623	361
		DSS	0.0197	1.7965	1.0979–2.9396	
		DFI	0.0208	1.4965	1.0631–2.1065	
		PFI	0.0254	1.4692	1.0485–2.0586	

## Discussion

LIHC is a leading malignant tumor worldwide with high metastasis and recurrence rate. Due to the complex molecular heterogeneity and poor prognosis of LIHC, it is urgent to develop biomarkers for LIHC prognosis. In the present study, we established a web server OSlihc using gene expression profiling and long-term clinical follow-up data. This prognosis analysis web server aims to assess the association between candidate gene and survival by an easy and interactive way. In current study, we have validated the association between survival and 65 previously published biomarkers for LIHC in our OSlihc server. As examples, three frequently-used biomarkers in LIHC, Osteopontin (OPN) (encoded by *SPP1* gene), Survivin (encoded by *BIRC5* gene) and Ki-67 (encoded by *MKI67* gene), showed prognostic abilities in OSlihc, and the elevated expression of these genes predicted the unfavorable prognosis, consistent with previous reports (King et al., 1998; Fields et al., 2004; Yang et al., 2008; Jin et al., 2014; Qin, 2014). These results demonstrated the reliability and capability of OSlihc web server in prognosis analysis.

The limitation of OSlihc is that only 637 LIHC clinical cases are currently available in OSlihc. When new datasets with profiling and clinical follow-up data become available, we will update the repository of OSlihc server to improve the performance.

In conclusion, we developed an online prognosis analysis tool named OSlihc, which provides a platform for researchers to discover the new prognostic biomarkers and may offer opportunities to develop novel targeted strategies for LIHC, conducing to clinical translation of potential biomarkers.

## Data Availability Statement

Publicly available datasets were analyzed in this study. This data can be found here: TCGA database (https://www.cancer.gov/about-nci/organization/ccg/research/structural-genomics/tcga) and GEO database (https://www.ncbi.nlm.nih.gov/gds/?term=).

## Author Contributions

YA, QW, and XG developed the server, performed the evaluation of novel prognostic biomarkers and drafted the paper. GZ, LZ, and FS performed the validation of previous reported biomarkers. HL, YL, and YP collected LIHC datasets. WZ and SJ contributed to data analysis and paper revision. All authors contributed to the article and approved the submitted version.

## Funding

This study is supported by National Natural Science Foundation of China (No.81602362), Supporting grants of Henan University (No.2015YBZR048, No.B2015151, No.2019YLXKJC04), Yellow River Scholar Program (No.H2016012), and Program for Innovative Talents of Science and Technology in Henan Province (No. 18HASTIT048), Program for Science and Technology Development in Henan Province (No.162102310391, No.172102210187), Program for Scientific and Technological Research of Henan Education Department (No.14B520022), Program for Young Key Teacher of Henan Province (No.2016GGJS-214), Kaifeng Science and Technology Major Project (No.18ZD008), Supporting grant of Bioinformatics Center of Henan University (No.2018YLJC01), and Innovation Project for College Students of Henan University (No.2019101904). All the funding sources have roles in study design, data collection, data analysis, and paper writing.

## Conflict of Interest

The authors declare that the research was conducted in the absence of any commercial or financial relationships that could be construed as a potential conflict of interest.

## Supplementary Material

Figure S1 Screenshot of OSlihc subfield interface. **(A)** TCGA, **(B)** GSE20140, **(C)** GSE76427 dataset (using BCLC stage as an example).

Figure S2 Validation of the top three high-frequency reported biomarkers in OSlihc. Kaplan–Meier plots for **(A–C)** *SPP1*, **(D–F)** *MKI67*, and **(G–I)** *BIRC5* in terms of DSS, DFI and PFI.

Figure S3 Gene expression analysis of *SPP1* **(A)**, *MKI67* **(B)** and *BIRC5* **(C)** at GEPIA with comparison between tumor and normal tissues.

Table S1 Clinicopathologic Characteristics of LIHC patients in TCGA dataset.

Table S2 Clinicopathologic Characteristics of LIHC patients in GEO datasets.

Table S3 Validation of previously published predictors for LIHC survival in OSlihc.

Table S4 Identification of novel potential prognostic biomarkers for LIHC in OSlihc.
