# Supplementary figures and images for: OSlihc: An Online Prognostic Biomarker Analysis Tool for Hepatocellular Carcinoma
Source: Front Pharmacol. 2020 Jun 10;11:875. doi: 10.3389/fphar.2020.00875 (PMC7298068; doi:10.3389/fphar.2020.00875)

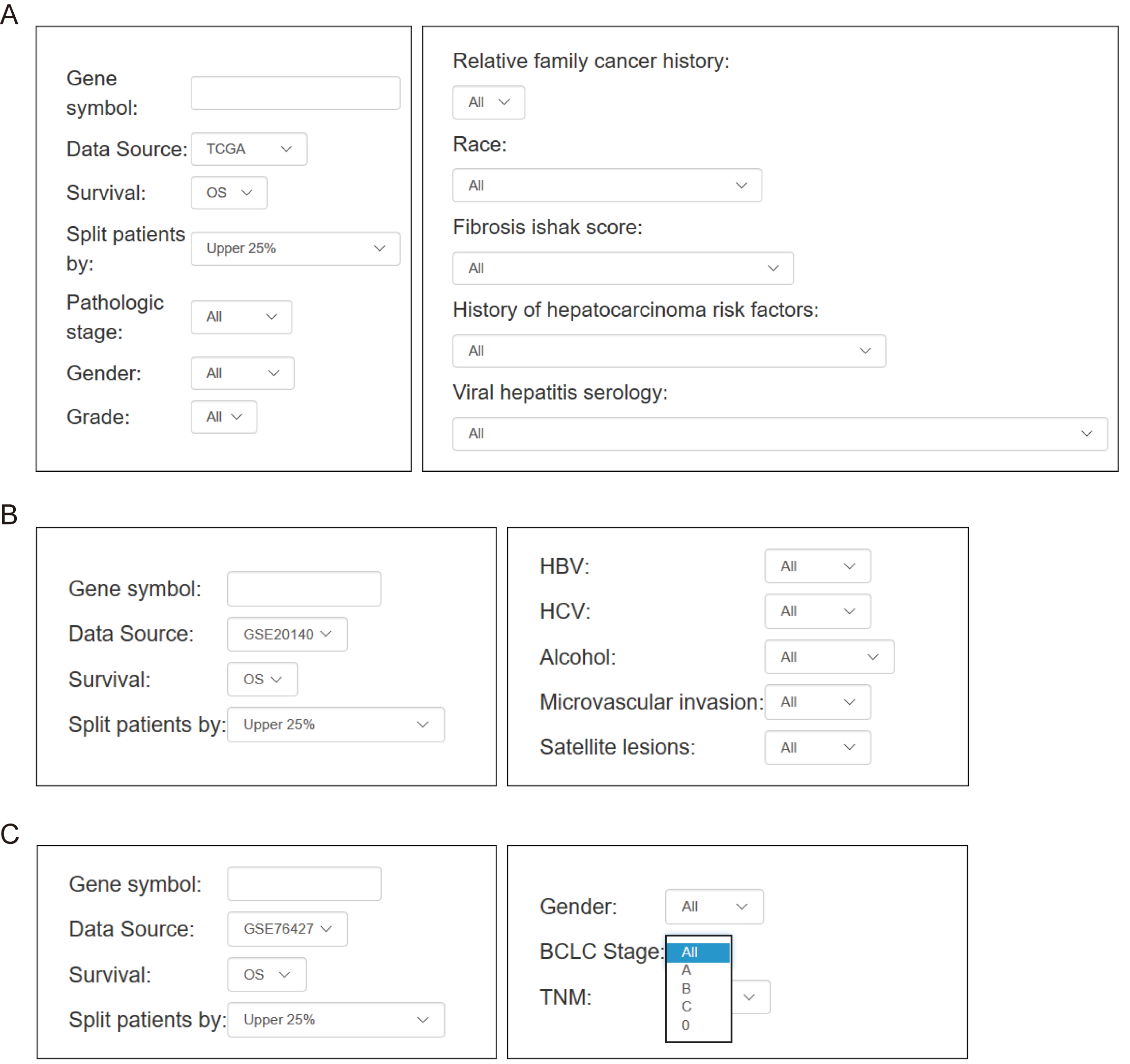

Supplement: Figure S1 — Screenshot of OSlihc subfield interface. (A) TCGA, (B) GSE20140, (C) GSE76427 dataset (using BCLC stage as an example). [file Image_1.tif]

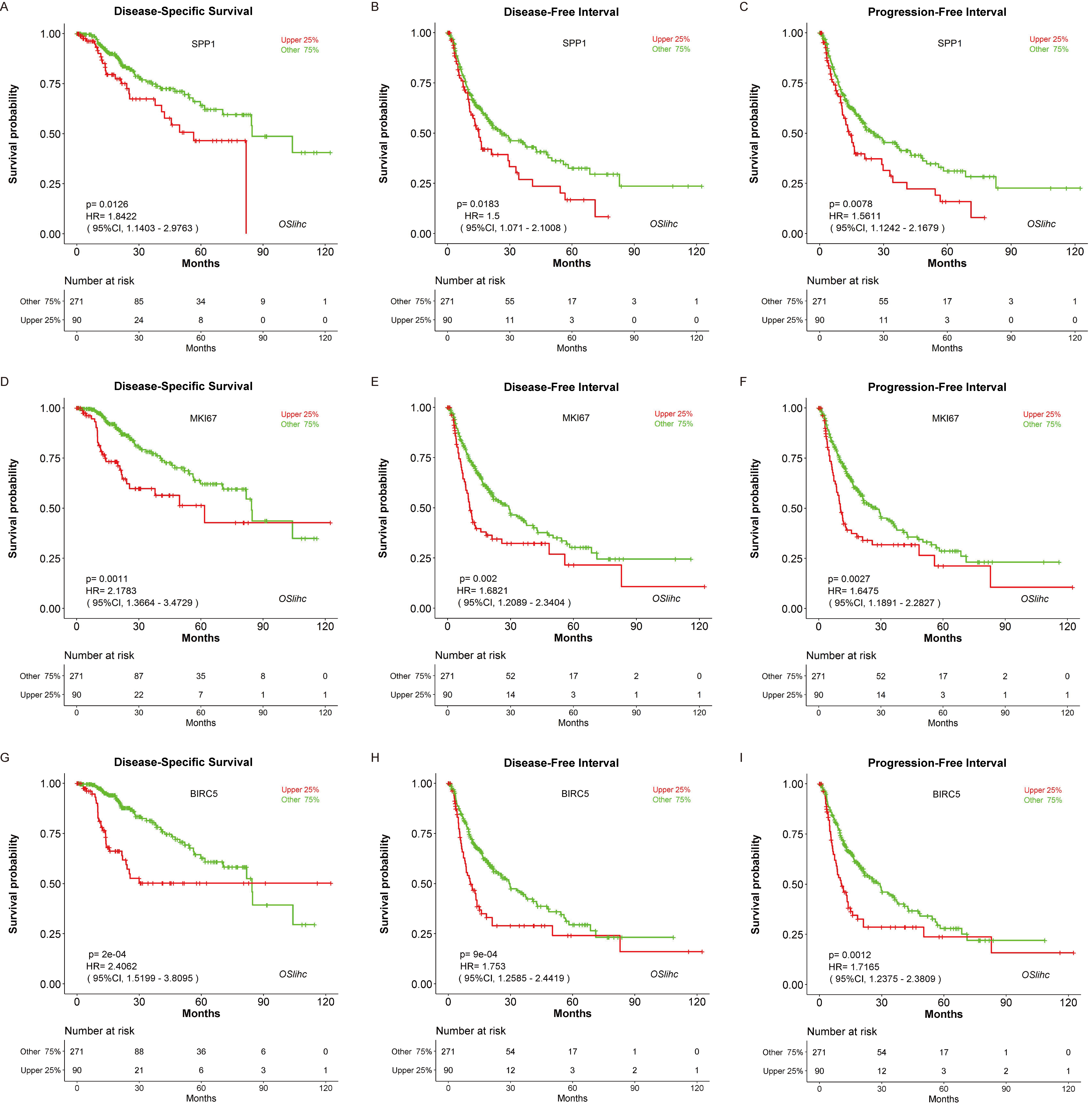

Supplement: Figure S2 — Validation of the top three high-frequency reported biomarkers in OSlihc. Kaplan–Meier plots for (A–C) SPP1, (D–F) MKI67, and (G–I) BIRC5 in terms of DSS, DFI and PFI. [file Image_2.tif]

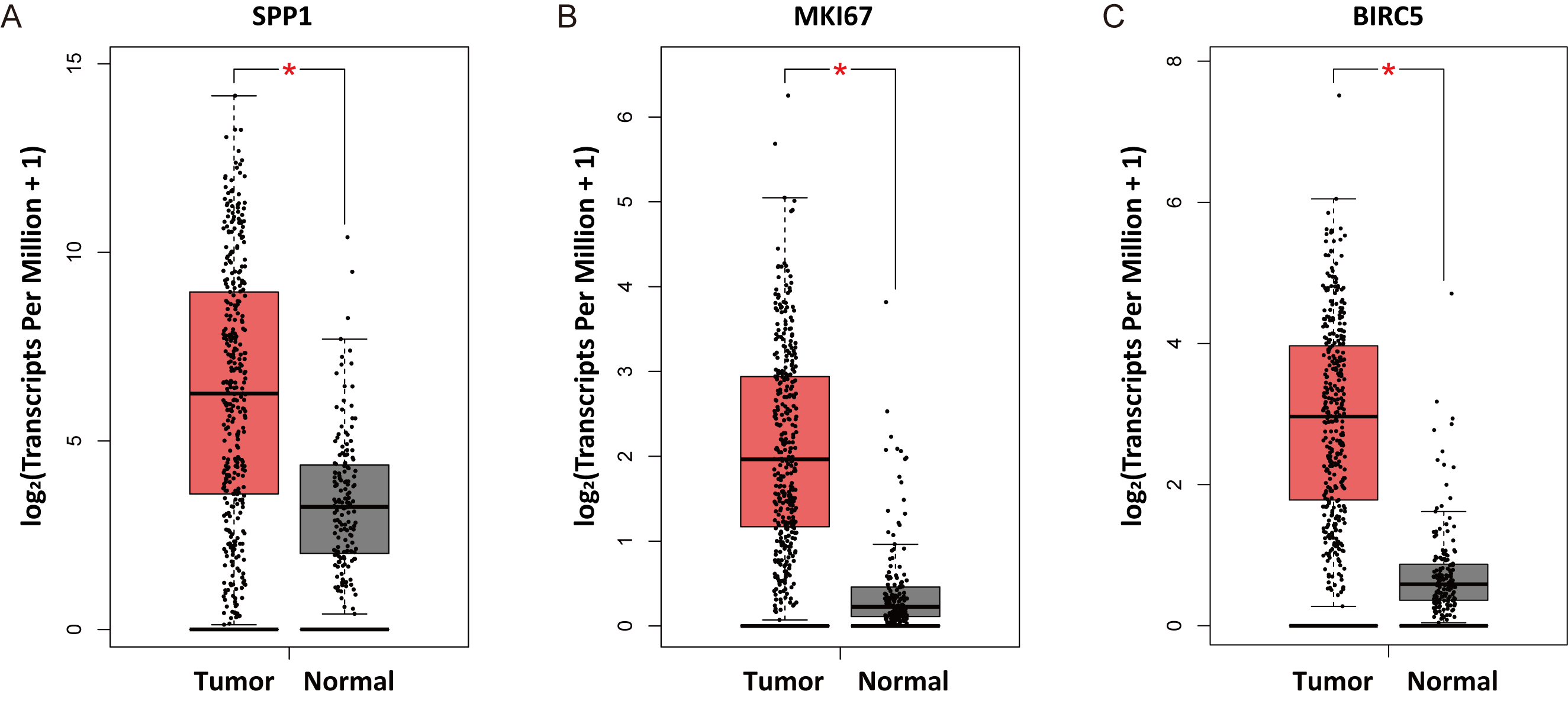

Supplement: Figure S3 — Gene expression analysis of SPP1 (A), MKI67 (B) and BIRC5 (C) at GEPIA with comparison between tumor and normal tissues. [file Image_3.tif]
